# MicroRNA transcriptomes of distinct human NK cell populations identify miR-362-5p as an essential regulator of NK cell function

**DOI:** 10.1038/srep09993

**Published:** 2015-04-24

**Authors:** Fang Ni, Chuang Guo, Rui Sun, Binqing Fu, Yue Yang, Lele Wu, Sitong Ren, Zhigang Tian, Haiming Wei

**Affiliations:** 1 Institute of Immunology and the CAS Key Laboratory of Innate Immunity and Chronic Disease, School of Life Science and Medical Center, University of Science and Technology of China, 443 Huang-Shan Road, Hefei 230027, China; 2 Department of Pathophysiology, Anhui Medical University, 81 Mei-Shan Road, Hefei, Anhui Province 230032, China; 3 Hefei National Laboratory for Physical Sciences at Microscale, 96 Jin-Zhai Road, Hefei, Anhui Province 230026, China

## Abstract

Natural killer (NK) cells are critical effectors in the immune response against malignancy and infection, and microRNAs (miRNAs) play important roles in NK cell biology. Here we examined miRNA profiles of human NK cells from different cell compartments (peripheral blood, cord blood, and uterine deciduas) and of NKT and T cells from peripheral blood, and we identified a novel miRNA, miR-362-5p, that is highly expressed in human peripheral blood NK (pNK) cells. We also demonstrated that CYLD, a negative regulator of NF-κB signaling, was a target of miR-362-5p in NK cells. Furthermore, we showed that the over-expression of miR-362-5p enhanced the expression of IFN-γ, perforin, granzyme-B, and CD107a in human primary NK cells, and we found that silencing CYLD with a small interfering RNA (siRNA) mirrored the effect of miR-362-5p over-expression. In contrast, the inhibition of miR-362-5p had the opposite effect in NK cells, which was abrogated by CYLD siRNA, suggesting that miR-362-5p promotes NK-cell function, at least in part, by the down-regulation of CYLD. These results provide a resource for studying the roles of miRNAs in human NK cell biology and contribute to a better understanding of the physiologic significance of miRNAs in the regulation of NK cell function.

NK cells play critical roles in the innate and adaptive immune responses during the early host defense against invading pathogens and tumors^1^,^2^,^3^,^4^. NK cells comprise up to 15% of all circulating lymphocytes and are also found in peripheral tissues, including the liver, lung, lymph nodes, and deciduas^5^. In humans, NK cells are identified as CD3*^−^* CD56^+^ lymphocytes without rearranged T-cell receptors and may be divided into CD56^bright^ and CD56^dim^ subsets based on the expression of CD56 and CD16 (Fc*γ*RIIIa)^6^. These subsets have different phenotypes, functions and tissue localizations^7^. Despite some additional characterizations of the phenotypic and functional differences between CD56^bright^ and CD56^dim^ NK cells, little is known about the molecular basis of the different phenotypes and functions of human NK cell subsets.

In response to activating stimuli, NK cells mediate several effector functions, including the production of cytokines, such as IFN-γ and TNF-a, as well as increased cytotoxicity against virus-infected and tumor cells^8^,^9^. Indeed, the roles of NK cells are diverse, and their functions are controlled by the balance of activating and inhibitory receptor signaling^10^,^11^. It is well known that NK-cell activation is regulated by intrinsic and extrinsic mechanisms that ensure NK tolerance and efficacy. Activation by exogenous cytokines, such as IL-2, IL-15, and IL-18, has been previously shown to positively regulate the cytotoxicity and IFN-γ production of NK cells, and endogenous nuclear transcription factors can also regulate NK cell functions. For example, STAT4 is required for Il-12-induced IFN-γ production and increased cytotoxicity^12^. IL-18 induces the nuclear localization of NF-kB p50/p65 that, together with AP-1, increases the cytotoxicity and IFN-γ production of NK cells^13^. Conversely, negative regulators (ATF3, Hlx, and Blimp1) have been reported to modulate the activation progress and IFN-γ secretion of NK cells^14^,^15^,^16^. Although many factors have been identified that contribute to key aspects of the NK cell molecular program, our understanding of the basic molecular mechanisms regulating NK cell effector functions (i.e., IFN-γ, perforin, and granzymes) is incomplete.

MicroRNAs (miRNAs) are highly conserved small non-coding single-stranded RNA molecules (19–24 nt) that act as key regulators of gene expression at the post-transcriptional level by binding to the 3′ untranslated region (UTR) of target mRNAs for translational repression or degradation^17^. These tiny regulators of gene expression have been shown to have unique expression profiles in the cells of the innate and adaptive immune systems and play pivotal roles in the regulation of development and the function of such cells^18^,^19^,^20^,^21^,^22^. Previous studies have measured the expression levels of miRNAs in selected immune cell systems, i.e., T cells^23^, B cells^24^, NK cells^25^, DCs^26^, and different lymphocyte subsets from peripheral blood mononuclear cells (PBMCs)^27^. Recently, increasing numbers of studies have shown that miRNAs play a critical role in controlling NK cell development, activation, and effector function^28^. We have previously demonstrated that miR-483-3p is preferentially expressed in human decidual NK (dNK) cells. We also revealed that endogenous IGF-1 is regulated by miR-483-3p in human NK cells and contributes to NK cell cytotoxicity^29^.

In this work, we shed light on the detailed miRNA expression profiles of 3 human NK populations isolated from different cell compartments (peripheral blood, cord blood, and uterine deciduas) and two other lymphocyte subsets (NKT cells and T cells from peripheral blood). Using this miRNA expression data as a starting point, we identified a novel miRNA, miR-362-5p, that was significantly up-regulated in pNK cells compared to that of the other lymphocyte subsets. Functional and mechanistic studies showed that miR-362-5p targets CYLD and regulates human NK cell function. Taken together, our results show that miR-362-5p plays an important role in human NK cell biology and suggest that the modulation of miR-362-5p may be a useful therapeutic strategy to regulate NK-cell effector function.

## Results

### Expression of miRNAs in primary human NK cells, NKT cells, and T cells

**Figure 1:**
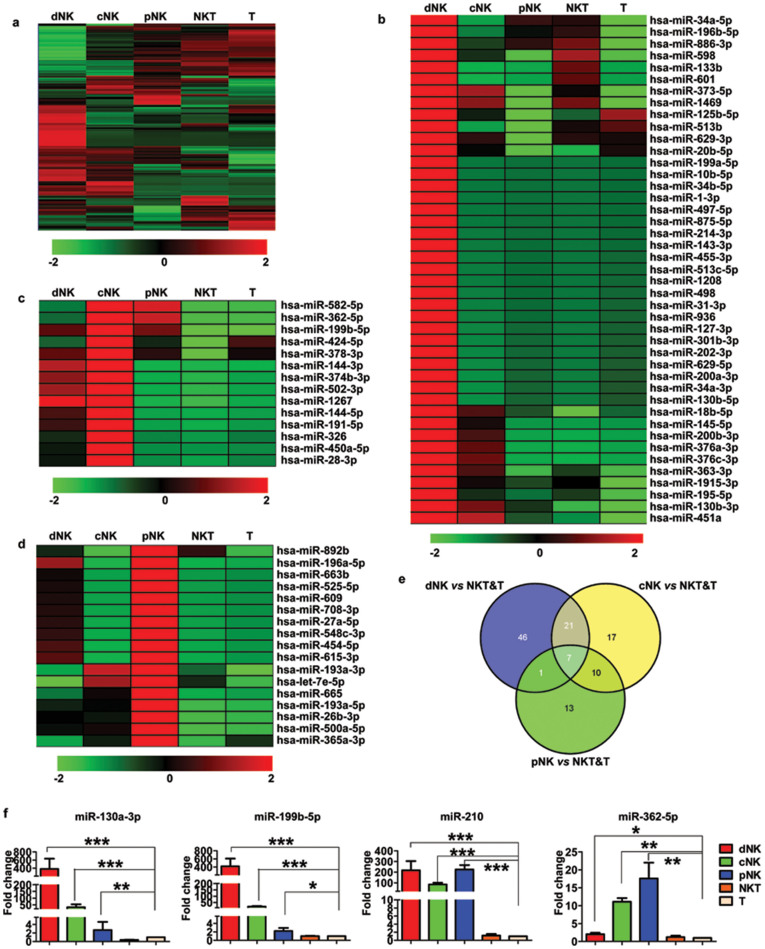
Expression profiles of miRNAs among different lymphocyte subsets. (a) Hierarchical cluster analysis of differentially expressed miRNAs in various cell subsets. (b–d) Heat map of the signature miRNAs for the dNK (b), cNK (c) and pNK (d) subsets: miRNAs with differences were selected by the expression with a fold change of two or greater in each subset when compared with the remaining four subsets. (e) The Venn diagram shows the number of all differentially expressed miRNAs across the following comparisons: dNK vs. NKT and T, cNK vs. NKT and T, and pNK vs. NKT and T. The number of differentially expressed miRNAs from each comparison is indicated. (f) Quantitative RT-PCR validation of the expression of miR-130a, miR-199b-5p, miR-210, and miR-362-5p in dNK, cNK, pNK, NKT, and T cells. RNU6B was used as an internal control for real-time PCR. Data are representative of three to six experiments. *P < 0.05, **P < 0.01, and ***P < 0.005 (Student's *t*-test).

In an effort to identify NK cell miRNA signatures that may contribute to an NK cell-specific molecular program, we determined the miRNA expression profiles of 3 various human NK cell populations and 2 other lymphocyte subsets (NKT and T cells). We initially purified different human NK cell subsets from the peripheral blood (pNK), cord blood (cNK) and uterine deciduas (dNK) as well as NKT and T cells from peripheral blood of healthy donors using flow cytometry sorting. The expression of 955 human miRNAs was determined in these highly purified human lymphocyte subsets. Hierarchical clustering indicated that each lymphocyte population was characterized by a distinct miRNA expression pattern (Fig. 1a). We also looked for miRNAs that were preferentially expressed in a single NK cell type and found that, relative to four other cell types, 43 miRNAs were two-fold upregulated in dNK cells (Fig. 1b and Supplementary Fig. S1A), 14 miRNAs were upregulated in cNK cells (Fig. 1c and Supplementary Fig. S1B) and 17 miRNAs were upregulated in pNK cells (Fig. 1d and Supplementary Fig. S1C).

We next searched for human NK cell-specific miRNAs in the entire data set. We selected miRNAs with a difference in expression of more than two fold in the given comparisons (dNK vs. NKT and T; cNK vs. NKT and T; pNK vs. NKT and T). These stringent criteria were met by 7 miRNAs that were specifically up-regulated in NK cells compared to NKT and T cells (miR-340-3p, miR-210, miR-199a-3p, miR-483-3p, miR-130a-3p, miR-199b-5p, and miR-362-5p) (Fig. 1e). To further verify the microarray data, 4 of these miRNAs were selected for real-time PCR validation. As shown in Fig. 1f, the data from the microarrays correlated well with the QRT-PCR results, although QRT-PCR generally showed larger fold changes than the microarray data.

### Differential miRNA expression analysis in various human NK populations

**Figure 2:**
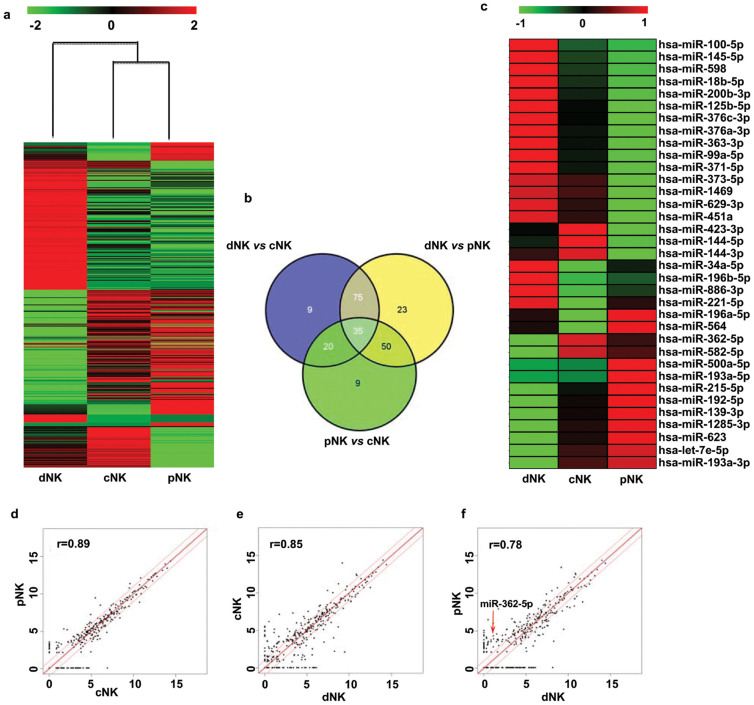
MiRNA profiles of various human NK populations. (a) Heat map of differentially expressed miRNAs in various human NK populations (dNK, cNK, and pNK). Each row represents an individual miRNA and each column represents an individual cell subset. Red, black, and green pseudocolors indicate transcripts levels below, equal, or above the mean, respectively, on a scale representing gene expression ratios from −2 to 2 on a log 2 scale. (b) The Venn diagram shows the number of all differentially expressed miRNAs across different comparisons (dNK vs. cNK, dNK vs. pNK, and cNK vs. pNK. The number of differentially expressed microRNAs from each comparison is indicated. (c) Heat map of the signature microRNAs with a fold change of two or greater in all three of the comparisons (dNK vs. pNK, dNK vs. cNK, cNK vs. pNK). Each row represents an individual miRNA, and each column represents an individual cell subset. Red, black, and green pseudocolors indicate transcripts levels below, equal, or above the mean, respectively, on a scale representing gene expression ratios from −1 to 1 on a log 2 scale. (d–f) Log base 2 intensity plots of miRNA levels for cNK vs. pNK samples (d), cNK vs. dNK samples (e), and dNK vs. pNK samples (f). The middle diagonal line represents equal expression, and the lines to each side represent 2-fold enrichment in either cell population. The labels of axes are log 2 scaled. Pearson correlation r values were used to establish the linear fit of the data.

In contrast to T and B lymphocytes, few studies have examined the miRNA expression profiles of human NK cells. We sought to determine whether NK cells from different cell compartments (e.g., peripheral blood, cord blood, and uterine deciduas) expressed different miRNAs. To further identify novel miRNAs that could provide clues to the differences between the various NK populations, we looked for differential microRNA expression in the different human NK cell populations (dNK, cNK, and pNK). Hierarchical clustering indicated that the miRNA profile of CD56^bright^ dNK cells was quite distinct from those of cNK and pNK cells (Fig. 2a). We also compared the miRNA profiles of all three NK subsets and selected the miRNAs with a difference in expression of more than two-fold in a given subset. Out of 955 miRNAs, Fig. 2b shows the number and distribution of 221 differentially expressed miRNAs across the different comparisons in the study. As suggested by the miRNA array data, miRNAs with a fold change of two or greater in all of the three comparisons (dNK vs. pNK; dNK vs. cNK; cNK vs. pNK) were considered. These stringent criteria were met by 35 miRNAs (Figs. 2b and 2c). To more closely examine differential miRNA expression in distinct NK populations (Fig. 2a), we performed pairwise comparisons between each of the two NK populations (cNK vs. pNK (d); dNK vs. cNK (e); and dNK vs. pNK (f)). Through Pearson correlation scatter plot analysis, we found that there was no significant difference in the expression levels of these miRNAs in cNK and pNK cells (r = 0.89, Pearson's correlation) (Fig. 2d), whereas the difference was more apparent between the dNK and pNK subsets (r = 0.78, Pearson's correlation) (Fig. 2f). These results indicated that the miRNA expression profiles of the cNK and pNK populations showed high similarities; however, the miRNA profile of dNK cells was distinct from that of the pNK population.

### MiR-362-5p is highly expressed in human pNK cells

**Figure 3:**
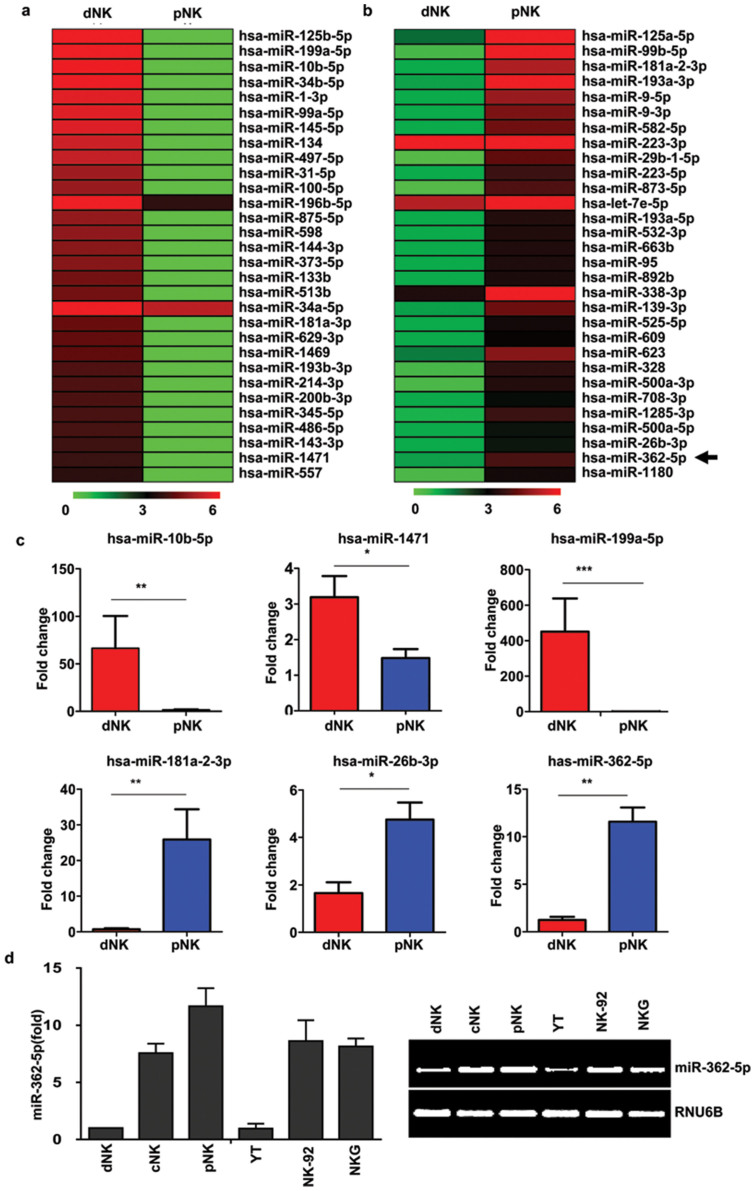
MiR-362-5p is highly expressed in human pNK cells. (a–b) miRNAs that were differentially expressed in dNK and pNK populations were divided into two groups consisting of (a) the top 30 miRNAs up-regulated in dNK cells and (b) the top 30 miRNAs up-regulated in pNK cells according to their distinct expression patterns based on hierarchical clustering. Each row represents an individual miRNA, and each column represents an individual cell subsets. Red and green pseudocolors indicate transcripts levels below or above the mean, respectively, on a log 2 scale representing gene expression ratios from 0 to 6. (c) Real-time PCR analysis of the expression of miR-10b-5p, miR-1471, miR-199a-5p, miR-181a-2-3p, miR-26b-3p, and miR-362-5p. (d) Quantitative RT-PCR analysis of miR-362-5p expression in various human primary NK cells and NK cell lines. The expression level was normalized to that of RNU6B. Data are representative of six independent experiments. *P < 0.05, **P < 0.01 and ***P < 0.005 (Student's *t*-test).

One of the goals of this work was to identify novel miRNAs that may contribute to the different phenotypes and functions of the CD56^bright^ dNK and pNK cells. To determine the relationship between these two subsets, hierarchical clustering was performed on differentially expressed miRNAs in the entire data set. Figs. 3a and 3b shows the top 30 miRNAs that were down- or upregulated in pNK cells compared with dNK cells. To validate the microarray data, we selected 3 miRNAs (miR-10b-5p, miR-1471 and miR-199a-5p) that were downregulated in pNK cells and 3 miRNAs (miR-181a-2-3p, miR-26b-3p and miR-362-5p) that were upregulated in human pNK cells for RT-PCR confirmation. Consistent with the microarray data, similar fold changes in the selected miRNAs were found with a high correlation between the microarray and PCR results (Fig. 3c).

An expanded analysis of 955 different miRNAs across 5 human lymphocyte subsets revealed that, although there were other differentially expressed miRNAs upregulated in NK cells compared with NKT and T cells (Figs. 1e and 1f), miR-362-5p was the only miRNA that was specifically upregulated by more than 10-fold in human pNK cells compared with dNK cells, a finding that was confirmed by qRT-PCR (Fig. 3d and Supplementary Fig. S2). Thus, we focused on miR-362-5p and hypothesized that this novel miRNA contributes to human NK cell biology.

### MiR-362-5p targets CYLD in human NK cells

**Figure 4:**
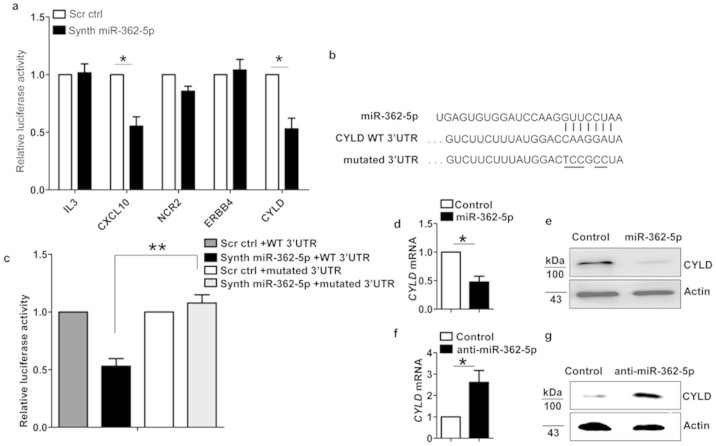
MiR-362-5p directly regulates *CYLD* expression in human NK cells. (a) Dual-luciferase assay of HEK-293T cells transfected with luciferase constructs containing genes (*n* = 5) predicted to be regulated by miR-362-5p, together with synthetic mature miR-362-5p (Synth miR-362-5p) or a synthetic control miRNA with scrambled sequence (Scr ctrl). (b) Diagram of the construction of wild-type (WT) or mutant CYLD 3′ UTR vectors. The mutant binding sequences are underlined. (c) Dual-luciferase assays of miR-362-5p co-transfected with luciferase constructs containing CYLD wild-type 3′ UTR (WT 3′ UTR) or mutated 3′ UTR into HEK 293T cells. The relative luciferase activity was normalized to the *Renilla* expression activity of the same vector. (d) Quantitative RT-PCR analysis of the expression of CYLD in dNK cells overexpressing miR-362-5p. (e) Western blot analysis of the expression of CYLD in dNK cells overexpressing miR-362-5p. Cropped blots are used. Full-length blots are presented in Supplementary Figure S7. Results are representative of three independent experiments. (f–g) Quantitative RT-PCR analysis (f), and Western blot analysis (g) of CYLD in sort-purified pNK cells transfected with FAM-labeled-miR-362-5p inhibitors (anti-miR-362-5p) or negative control miRNA. Full-length blots are presented in Supplementary Figure S7. Data are from three independent experiments with similar results. *P < 0.05, **P < 0.01 (Student's *t*-test).

Because miRNAs function primarily through the inhibition of target genes, we next sought to identify the mRNA targets regulated by miR-362-5p in human NK cells that might be involved in human NK cell function. We used TargetScan to make bio-informative predictions of the potential miR-362-5p target genes. We assessed the inhibitory effect of miR-362-5p on 5 putative target genes relative to NK cell function. We cloned the 3′ UTRs of these genes into the psi-CHECK2 vector and co-transfected the resulting vectors into HEK293T human epithelial cells along with synthetic mature miR-362-5p double-stranded RNA (Synth miR-362-5p) or a control miRNA with a scrambled sequence (Scr ctrl). We found that miR-362-5p directly regulated 2 of the 5 3′ UTRs (Fig. 4a). One candidate identified by this approach was the cylindromatosis (CYLD) gene. We further demonstrated that mutation of the miR-362-5p-responsive elements in the CYLD 3′ UTR resulted in the abrogation of the inhibitory effect of miR-362-5p (Figs. 4b and 4c). CYLD is a deubiquitination enzyme that plays a predominant role in the negative regulation of the nuclear factor kappa B (NF-κB) pathway. Because NF-κB has been proven to play critical roles in the regulation of NK cell activity and cytokine production^30^,^31^,^32^,^33^, we hypothesized that miR-362-5p might inhibit CYLD expression to induce downstream NF-κB signaling, thereby regulating NK cell function.

To experimentally verify CYLD as a target of miR-362-5p in human NK cells, we examined the effect of miR-362-5p overexpression on endogenous CYLD expression. Because miR-362-5p was downmodulated in human primary dNK cells (Figs. 1f and 3d), we assessed the direct regulation of CYLD expression as a result of miR-362-5p overexpression in dNK cells. Figs. 4d and 4e indicate a significant reduction in CYLD mRNA and protein levels in human dNK cells after miR-362-5p overexpression. Because miR-362-5p was upregulated in human primary pNK cells (Fig. 3d), we used nucleofection to knock down miR-362-5p with miR-362-5p inhibitors and measured CYLD expression in pNK cells. The knockdown of miR-362-5p led to a substantial increase in CYLD mRNA and protein levels in human primary pNK cells (Figs. 4f and 4g). Collectively, the above results suggest that miR-362-5p directly targets CYLD in human NK cells.

### Overexpression of miR-362-5p promotes human NK cell effector function

**Figure 5:**
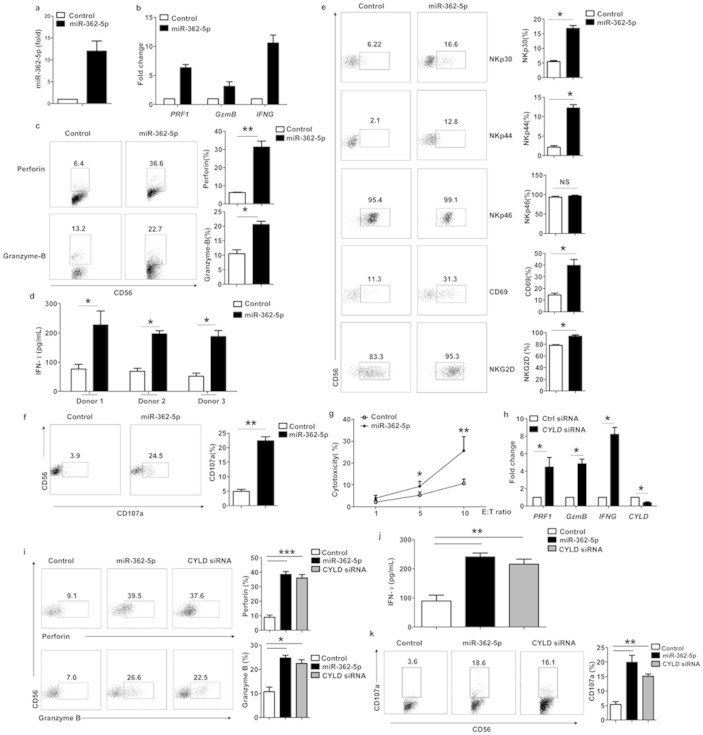
Overexpression of miR-362-5p promotes human NK cell effector function. (a–b) Quantitative RT-PCR assay of miR-362-5p (a) and *PRF1*, *GzmB*, and *IFNG* (b) in dNK cells transfected with miR-362-5p mimics. (c) Flow cytometry analysis of the expression of perforin, granzyme-B in purified human dNK cells transfected with miR-362-5p mimics or miRNA with scrambled sequence (Control). The graphs show the average relative frequency of all perforin^+^ or granzyme B^+^ dNK cells. (d) ELISA of IFN-γ in the supernatants of purified dNK cells transfected with miR-362-5p mimics or control miRNA that were stimulated overnight with IL-2 (100 U/ml), IL-12 (10 ng/ml), and IL-18 (100 ng/ml), beginning 20 h after transfection. Data represent mean of three independent wells. *P < 0.05 among all three donors for control versus miR-362-5p. (e) Flow cytometry analysis of the surface expression of NKp30, NKp44, NKp46, CD69, and NKG2D in dNK cells in c. The graphs show the average relative frequency of all NKp30^+^, NKp44^+^, NKp46^+^, CD69^+^, and NKG2D^+^ dNK cells. (F) Flow cytometry for CD107a expression in dNK in c. The graphs show the average relative frequency of CD107a^+^ dNK cells as above. (g) Flow cytometry assay evaluating the cytotoxic activity of dNK cells in c. Results are expressed as mean ± SEM of triplicate wells from one representative experiment of three experiments completed. (h) Quantitative RT-PCR analysis of *PRF1,* *GzmB*, *IFNG*, and *CYLD* expression in dNK cells transfected with *CYLD* siRNA or control siRNA (Ctrl siRNA). Data are representative of three independent experiments with similar results. (i) Intracellular staining of perforin and granzyme-B in purified dNK cells transfected by nucleofection with miR-362-5p mimics, negative control, or CYLD siRNA. (j) ELISA of IFN-γ in the supernatants of purified dNK cells in I. that were stimulated overnight with IL-2 (100 U/ml), IL-12 (10 ng/ml), and IL-18 (100 ng/ml), beginning 20 h after transfection. (K) Flow cytometry for CD107a expression in purified dNK cells in i. Data are representative of three independent experiments (mean ± SEM). *P < 0.05, **P < 0.01 and ***P < 0.005 (Student's *t*-test).

Next, we studied the functional role of miR-362-5p in modulating human NK cell function by a gain-of-function approach. Purified human dNK cells transfected with miR-362-5p mimics expressed substantially more miR-362-5p than cells transfected with negative control miRNA (Fig. 5a). Because cytotoxicity and cytokine production are major functions of NK cells, we investigated the levels of the cytotoxic effector genes perforin and granzyme B and of the cytokine interferon-γ (IFN-γ) to determine whether NK cell effector function was affected by the increased miR-362-5p expression. Compared with the negative control miRNA, the use of nucleofection to upregulate miR-362-5p with miR-362-5p mimics caused a significant increase in effector function in human dNK cells, as demonstrated by their higher production of perforin, granzyme B and IFN-γ (Figs. 5b, 5c and 5d). We also used flow cytometric analysis to measure the surface expression of NKp30, NKp44, NKp46, CD69 and NKG2D on dNK cells. The expression levels of the examined receptors were almost all increased in dNK cells transfected with miR-362-5p mimics compared with the control cells (Fig. 5e).

We next investigated the influence of miR-362-5p on the degranulation of human NK cells. Although degranulation is just one step in the NK cell killing process, the expression of CD107a on the cell membrane correlates well with NK cell cytotoxicity^34^. We found that the overexpression of miR-362-5p resulted in significantly increased CD107a expression (Fig. 5f). To further assess whether miR-362-5p regulates cytotoxicity, purified dNK cells were transfected with either a miR-362-5p mimic or a control miRNA for 20 h. Cytotoxicity against the K562 leukemia cell line was assessed by FACS. The overexpression of miR-362-5p resulted in a substantial increase in the cytotoxic activity of dNK cells (Fig. 5g). Overall, these data suggest that miR-362-5p is a critical positive regulator of NK cell function.

A corollary of our hypothesis that miR-362-5p promotes human NK cell effector function by targeting CYLD is that knockdown of CYLD will enhance NK cell function. Following this reasoning, we used RNA interference technology to successfully knock down CYLD expression, as determined by QRT-PCR analysis (Fig. 5h). Purified dNK cells were transfected with either scramble control siRNA or CYLD siRNA. The knockdown of CYLD led to an increase in NK cell function compared with the scramble controls, mirroring the phenotype observed with the overexpression of miR-362-5p (Figs. 5i, 5j and 5k).

We demonstrated above that the overexpression of miR-362-5p promotes human NK cell effector function in human NK cells. In addition, it has been demonstrated that NF-κB plays critical roles in the regulation of NK cell activity and cytokine production^30^,^31^,^32^,^33^. To further illustrate the mechanism underlying the positive regulation of NK cell effector function by miR-362-5p, we examined the impact of miR-362-5p on NF-κB activation by detecting the level of nucleus NF-κB P65 protein in human dNK cells transfected with miR-362-5p mimics. The results indicated that the level of nuclear NF-κB P65 protein increased (Supplementary Fig. S3). To ascertain whether the NF-κB signaling pathway is responsible for the increased function of miR-362-5p-transfected NK cells, we pretreated control and miR-362-5p-transfected dNK cells with pyrrolidine dithiocarbamate (PDTC, a specific inhibitor of NF-κB) before cytokine stimulation. As shown in Supplementary Fig. S4, miR-362-5p overexpression dramatically enhanced the cytokine-induced IFN-γ production of dNK cells, and this enhancement was attenuated by PDTC. Taken together, these results support the conclusion that miR-362-5p promotes human NK function, at least in part, through increased activation of the NF-κB pathway.

### Inhibition of miR-362-5p acts through CYLD to suppress human NK cell function

**Figure 6:**
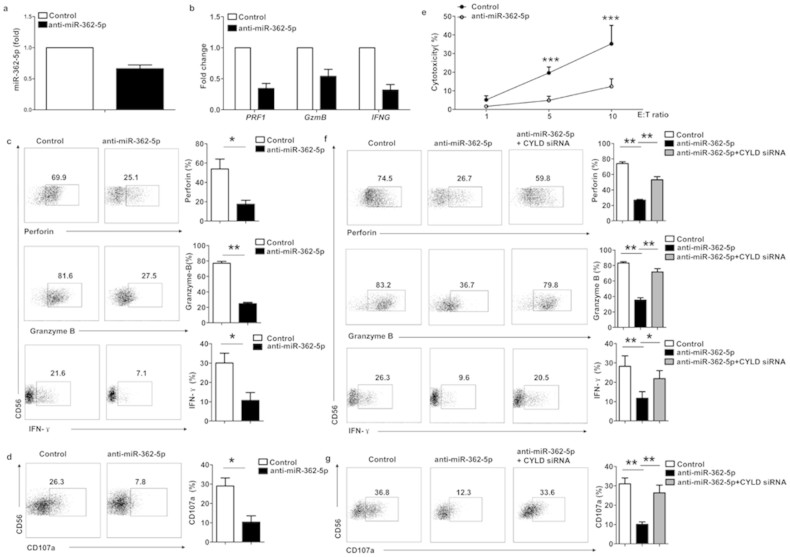
Inhibition of miR-362-5p acts through CYLD to suppress human NK cell function. (a–b) Quantitative RT-PCR assay of miR-362-5p (a), and *PRF1*, *GzmB*, and *IFNG* (b) in sort-purified pNK cells transfected with synthetic FAM-labeled-miR-362-5p inhibitor (anti-miR-362-5p) or a synthetic control miRNA (Control). (c–d) Flow cytometry of the expression of perforin, granzyme-B, and IFN-γ; (c), and CD107a (d) in purified human pNK cells transfected with FAM-labeled-anti-miR-362-5p or negative control miRNA (Control). FAM positive pNK cells were gated and analyzed. The graphs show the average relative frequency of perforin^+^, granzyme-B^+^, IFN-γ, or CD107a^+^ pNK cells as determined above. (e) Flow cytometry assay evaluating the cytotoxic activity of pNK cells transfected with anti-miR-362-5p or control miRNA. Results are expressed as mean ± SEM of triplicate wells from one representative experiment of three experiments completed. (f–g) Flow cytometry analysis of the expression of perforin, granzyme-B and IFN-γ (f); and CD107a (g) in purified pNK cells transfected with FAM-anti-miR-362-5p in the presence or absence of CYLD siRNA. Representative FACS plots from FAM^+^ cells are shown. The graphs show the average relative frequency of all perforin^+^, granzyme-B^+^, IFN-γ^+^, or CD107a^+^ pNK cells as above. Data are representative of three independent experiments (mean ± SEM). *P < 0.05, **P < 0.01 and ***P < 0.005 (Student's *t*-test).

To investigate the physiological importance of endogenous miR-362-5p in the regulation of NK cell function, we assessed the effect of miR-362-5p loss-of-function in human primary pNK cells. Following the nucleofection-mediated transfection of primary pNK cells with an FAM-labelled miR-362-5p inhibitor, the expression of miR-362-5p was downregulated to some degree in the miR-362-5p inhibitor-transfected pNK cells (Fig. 6a). Furthermore, we found by quantitative real-time PCR and flow cytometric analysis that the expression of perforin, granzyme-B, and IFN-γ was also significantly decreased in the above miR-362-5p inhibitor-transfected pNK cells compared with the pNK cells transfected with a control inhibitor (synthetic RNA with a random sequence; Figs. 6b and 6c). Next, we examined the effect of miR-362-5p loss of function on the cytotoxic activity of human NK cells. When transfected with a miR-362-5p inhibitor (anti-miR-362-5p), human primary pNK cells showed a dramatic decrease in their cytotoxic activity and a CD107a release (Figs. 6d and 6e).

We hypothesized that increased CYLD levels may mediate the inhibitory effect of miR-362-5p. To confirm our hypothesis, we transfected CYLD siRNA into the anti-miR-362-5p-transfected pNK cells to knockdown CYLD expression. Indeed, CYLD siRNA reversed the decrease in perforin, granzyme B, IFN-γ and CD107a expression (Figs. 6f and 6g). These results suggested that miR-362-5p acts through the CYLD pathway to regulate NK cell function.

## Discussion

The functions of miRNAs in immune cell development and function have attracted much attention and research interest. An increasing number of studies indicate that specific miRNAs can play key roles in controlling NK cell homeostasis, activation, and effector function, in addition to regulating diverse aspects of NK cell biology^28^. In the present study, we performed miRNA expression profiling for 5 human immune cell subsets (dNK. cNK, pNK, NKT, and T cells) to identify human NK cell-type-specific miRNAs. Our results indicated that the NK cells had a far greater number of significantly differentially expressed miRNAs than NKT or T cells. Comparisons of the expression patterns among the three NK populations (dNK, cNK and pNK) provided further insight. Furthermore, we used qRT-PCR to verify that miR-362-5p was highly specific for human pNK cells. Moreover, we demonstrated that miR-362-5p acts as a positive regulator of NK cell function by upregulating the expression of IFN-γ, granzyme-B, and perforin and enhancing the degranulation of NK cells in humans.

Previous oligonucleotide microarray data indicate that pNK and dNK cells differ in their patterns of gene expression^35^. However, no comprehensive studies of miRNA profiles across different human primary NK populations have been reported. In our previous studies, to find candidate regulators of the IGF-1 gene in human NK cells, we assessed miRNA expression in human primary lymphocytes (dNK, cNK, pNK, NKT, and T cells) with miRNA microarray analysis, and found that miR-483-3p functions as a critical regulator of human NK cell cytotoxicity by directly targeting IGF-1^29^. Additionally, these results may affect the biological and physiological significance of this human NK cell miRNA transcriptomes. In the present study, to identify novel miRNA candidates in human NK cells and investigate the roles of miRNAs in human NK cell activation and function, we performed a broad and deep re-analysis of miRNA expression in 5 different highly purified human lymphocyte subsets (dNK, cNK, pNK, NKT, and T cells) with that previously unpublished array data. In particular, we focused on the miRNAs upregulated in different human primary NK cells (dNK, cNK and pNK cells). The large number of novel miRNAs that were identified in this study offer new research targets for a more complete understanding of human NK cell biology.

The comparative analysis of the miRNAs expressed by dNK cells versus those expressed by pNK cells revealed intriguing differences in their miRNA expression profiles. It is not possible to fully elucidate the biological differences that account for such variation with this type of miRNA array-based study. However, our data analysis identified several miRNAs, such as miR-362-5p, that were up-regulated in pNK cells and are associated with the function of human NK cells. This observation suggests that concordant differences in miRNA expression levels may contribute to the differences between CD56^bright^ dNK and CD56^dim^ pNK subsets.

Our data suggest that miR-362-5p promotes the effector function of human NK cells, at least in part, through the down-regulation of CYLD. CYLD was identified as a tumor suppressor with deubiquitinase activity^30^. Previous studies have revealed multiple roles for CYLD in immune cell development and homeostasis using CYLD-deficient mice^36^,^37^,^38^,^39^,^40^,^41^. However, little is known about the role of CYLD in human NK cells. In this study, we showed that knockdown of CYLD by siRNA mirrored the effect of miR-362-5p over-expression and resulted in significantly increased perforin, granzyme B, IFN-γ and CD107a expression in human NK cells. Therefore, these findings establish CYLD as a critical regulator of human NK cell function and provide molecular insights into this novel function of CYLD.

CYLD has been shown to negatively regulate the activation of NF-κB^42^. Because NF-κB signaling is important in NK cell function^31^,^32^,^33^ and because CYLD is a direct target of miR-362-5p, we hypothesized that miR-362-5p likely regulates human NK cell function through the CYLD-NF-κB pathway. Although we do not have direct evidence that miR-362-5p influences NK cell function through NF-κB signaling pathway, this hypothesis is supported by the observation that IFN-γ production was significantly decreased by an NF-κB inhibitor in both control and miR-362-5p overexpressing NK cells. Thus, it is likely that miR-362-5p enhances IFN-γ production in NK cells by regulating the NF-κB pathway.

In summary, the miRNA transcriptomes of human dNK, cNK, and pNK cells are presented in this study, thus opening avenues for future research and bringing the phenotypic and functional differences between the distinct NK populations into clearer focus. In addition, we revealed that a novel miRNA, miR-362-5p, was highly expressed in human pNK cells where it promoted NK cell effector function by targeting CYLD. Our data offer a new view of human NK cells and provide resources for investigating the roles of miRNAs in human NK cell biology.

## Methods

### Ethics statement

This study was approved by the Medical Ethics Committee of the University of Science and Technology of China. All human subjects and experimental protocol were performed according to the approved guidelines established by the Institutional Human Research Subjects Protection Committee of the Ethics Committee of the University of Science and Technology of China. All samples were obtained after donor informed consent.

### Samples and cell lines

All decidual samples from patients undergoing elective abortion in the first trimester between 6 and 12 weeks of gestation were collected at Hefei Maternity and Child Care Hospital. Cord blood samples from the umbilical cords of the placentas of normal, full-term, non-stressed newborns of consenting mothers were also collected at Hefei Maternity and Child Care Hospital. Adult peripheral blood samples were obtained from healthy donors at Hefei Blood Bank. The NK-92 cell lines were grown in α-MEM (Life Technologies) medium supplemented with 15% heat-inactivated fetal bovine serum (FBS) (Hyclone), 15% horse serum, 100U/ml IL-2 and 100U/ml streptomycin/penicillin. The YT cell line, NKG cell line, and human K562 erythroleukemia cells were cultured in RPMI-1640 medium (Gibco BRL) containing 10% heat-inactivated FBS and 100U/ml streptomycin/penicillin. The HEK293T cells were cultured in DMEM medium (Gibco BRL) supplemented with 10% FBS.

### Isolation of human NK cells, T cells, and NKT cells

The cells were processed within 4 h of collection. Blood samples were diluted 1:2 in PBS. Mononuclear cells were isolated by Ficoll-Hypaque centrifugation using standard procedures. In some experiments, CD3^−^CD56^+^ NK cells were isolated from cord blood mononuclear cells with the MACS isolation system according to the manufacturer's instructions (Miltenyi Biotec). NK (CD3^−^CD56^+^), T (CD3^+^CD56^−^) and NKT (CD3^+^CD56^+^) lymphocyte subsets were purified from peripheral blood mononuclear cells by sorting on a FACSAria (BD) according to various combinations of surface markers. Decidual NK cells were isolated from decidual samples as previously described^35^. The cell purity was > 95% by post-FACS analysis (Supplementary Fig. S5).

### MicroRNA expression profiling

The miRNA expression profiles of normal human NK cells, cNK cells, pNK cells, T and NKT cells were investigated with the Human miRNA Microarray kit v12.0 (Agilent Technologies, Santa Clara, CA), which allows for the detection of 955 known human miRNAs (miRBase v12.0), as previously described^43^. Six mixed samples for each of dNK cells, cNK cell, pNK cell, NKT cells, and T cells were hybridized on the arrays. The integrity of these total RNAs was assessed using an Agilent 2100 Bioanalyzer, the total RNA was labeled with the miRNA Complete Labeling and Hyb Kit (Agilent Technologies), and fluorescent signals were extracted and analyzed with Feature Extraction Software (version 9.5.3.1, Agilent Technologies). GEO accession number: GSE66325.

### RNA isolation and real-time PCR analysis

Total RNA was isolated with TRIzol Reagent (Invitrogen) exactly as previously described^29^. For miRNA analysis, real-time PCR was performed according to the manufacturer's instructions (Qiagen, miScript Reverse Transcription Kit, miScript Primer Assay, and miScript SYBR Green PCR Kit). The small nucleolar RNA RNU6B was used as an internal control. For mRNA analysis, the total RNA was reverse-transcribed using Moloney murine leukemia virus reverse transcriptase (Invitrogen) and random primers (Roche). Real-time PCR was performed using SYBR Premix Ex Taq II (TaKaRa, China) according to the manufacturer's instructions. The GAPDH mRNA level was used as an internal normalization control. The following primers were used to detect mRNA expression:

CYLD:5′ – TCAGGCTTATGGAGCCAAGAA – 3′ (forward), and

5′ – ACTTCCCTTCGGTACTTTAAGGA – 3′ (reverse).

GAPDH: 5′ – ATGGGGAAGGTGAAGGTCG – 3′ (forward), and

5′ – GGGGTCATTGATGGCAACAATA – 3′ (reverse).

### Western blot analysis

Cells were lysed in RIPA buffer supplemented with protease inhibitors (Pierce). After centrifugation at 13,000 × *g* for 5 minutes at 4°C to remove cell debris, the pellet was discarded. Equal amounts of protein were separated by SDS-PAGE and subsequently transferred onto PVDF membranes (Millipore). CYLD was detected with a mouse anti-CYLD monoclonal antibody (Santa Cruz Biotechnology) overnight at 4°C. The membrane was then incubated with a goat anti-mouse IgG, horseradish peroxidase-linked secondary antibody. Immuno-reactive protein bands were developed by chemiluminescence autoradiography.

### Dual-luciferase reporter assay

A fragment of the 3′ UTR of CYLD DNA containing the wild-type or mutated target sequence of miR-362-5p was inserted into the digested psiCHECK-2 vectors (Promega). The inserted sequences were confirmed by sequencing. The HEK293T cells were transfected with 10 ng each of psi-CHECK-2 construct along with 15 pmol of the miR-362-5p mimic or an identical amount of the negative control miRNA using Lipofectamine 2000 (Invitrogen). Assays were performed 48 h after transfection using the Dual-Luciferase Reporter Assay System (Promega). The results of the relative luciferase activity were presented as the ratio of *Renilla* luciferase activity to firefly luciferase activity.

### Nucleofection of human NK cells

Synthetic miR-362-5p mimics, miR-362-5p inhibitors, CYLD siRNAs, and negative controls were all purchased from GenePharma. An Amaxa Human NK Cell Nucleofector Kit (VPA-1005) was used for the transfection of human primary NK cells. The transfection efficiency of the FAM-labeled-miR-362-5p mimics or inhibitors in human NK cells is shown in Supplementary Fig. S6. Cells were plated in complete medium and subjected to FACS, RT-PCR, or western blot analysis.

### Flow cytometry and intracellular staining

The following antibodies were used for flow cytometry analysis: anti-CD3 conjugated with PE-Cy5; anti-CD56 conjugated with Alexa-647; PE-conjugated anti-NKp30, anti-NKp44, anti-NKp46, anti-CD69, anti-NKG2D, anti-granzyme B, anti-IFN-γ, anti-perforin and anti-CD107a; and isotype-matched negative control antibodies labeled with PE, PE-Cy5, or Alexa-647 (BD Pharmingen). For surface receptor staining, NK cells were stained with specific antibodies. For intracellular perforin and granzyme-B staining, NK cells were stained using the indicated Abs with the Fixation/Permeabilization Solution Kit (BD Pharmingen), according to the manufacturer's instructions. For the intracellular detection of IFN-γ, NK cells were stimulated overnight with IL-2 (100 U/ml, PeproTech), IL-12 (10 ng/ml, PeproTech), and IL-18 (100 ng/ml, PeproTech). Cells were then analyzed for the production of IFN-γ.

### NK cell degranulation

NK cell degranulation was measured by flow cytometry as previously described^44^. Briefly, 3 × 10^5^ purified human NK cells and 3 × 10^5^ K562 leukemia cells were mixed by gentle pipetting, spun down for 1 min at 1000 rpm, and incubated for 2 h at 37°C in 200 µl of media as indicated. Cells were then surface labeled with an anti-CD56-Alexa-647 antibody, and degranulation was detected with an anti-CD107a-PE antibody (all antibodies from BD Biosciences). Cells were washed and fixed in 4% paraformaldehyde before being analyzed by FACS.

### Assay of NK cell cytotoxicity

NK cell cytotoxicity was analyzed using flow cytometry as previously described with slight modifications^45^. Briefly, human NK cells were incubated with K562 in different E: T ratios for 12 h at 37°C and 5% CO_2_. NK cell cytotoxicity against K562 cells was measured using three-color flow cytometry. At the indicated time points, cells were collected and incubated with anti-CD56 and annexin V. After washing, the cells were stained with 7-aminoactinomycin D (7-AAD) and analyzed by FACS. Live target cells were determined by gating on the CD56^−^annexin V^−^7-AAD^−^ population. The percentage of cytotoxicity was calculated by the following equation: 100 × (CD56^−^ cells − CD56^−^ annexin V^−^ 7-AAD^−^ cells)/(CD56^−^ cells).

### ELISA

The IFN-γ concentrations in NK cell culture supernatants were quantified using an ELISA kit, according to the manufacturer's instructions (R&D Systems).

### Statistical analysis

Data were analyzed by two-sided non-parametric Student's *t*-test, unless otherwise indicated. For analysis of more than two groups, a one-way analysis of variance (ANOVA) was used. A P-value of 0.05 or less was considered significant, and the degree of significance is indicated as follows: *, P < 0.05; **, P < 0.01, and ***P < 0.005.

## Author Contributions

F.N. designed and performed experiments, analyzed data and wrote the manuscript; C.G. established techniques for flow cytometry and performed the bioinformatic and statistical analyses; R.S and B.F. discussed results, provided professional advice and commented on the manuscript; Y.Y., L.W. and S. R. contributed materials and/or analytical tools. Z.T & H.W designed the study, supervised research and revised the manuscript. All authors reviewed the manuscript.

## Supplementary Material

Supplementary Information Supplementary figures
