# Supplementary material for: MicroRNA transcriptomes of distinct human NK cell populations identify miR-362-5p as an essential regulator of NK cell function
Source: Sci Rep. 2015 Apr 24;5:9993. doi: 10.1038/srep09993 (PMC4408982; doi:10.1038/srep09993)
Supplement: Supplementary Information — Supplementary figures [file srep09993-s1.pdf]

Supplementary Information

MicroRNA transcriptomes of distinct human NK cell populations identify miR-362-5p as an essential regulator of NK cell function

Fang Ni[1,2 *], Chuang Guo[1*], Rui Sun[1], Binqing Fu[1,3], Yue Yang[2], Lele Wu[2], Sitong Ren[2], Zhigang Tian[1,3] & Haiming Wei[1]

Affiliations

[1]Institute of Immunology and the CAS Key Laboratory of Innate Immunity and Chronic Disease, School of Life Science and Medical Center, University of Science and Technology of China, 443 Huang-Shan Road , Hefei 230027, China.

[2]Department of Pathophysiology, Anhui Medical University, 81 Mei-Shan Road, Hefei, Anhui Province 230032, China

[3]Hefei National Laboratory for Physical Sciences at Microscale，96 Jin-Zhai Road, Hefei, Anhui Province 230026, China

Supplementary Figures

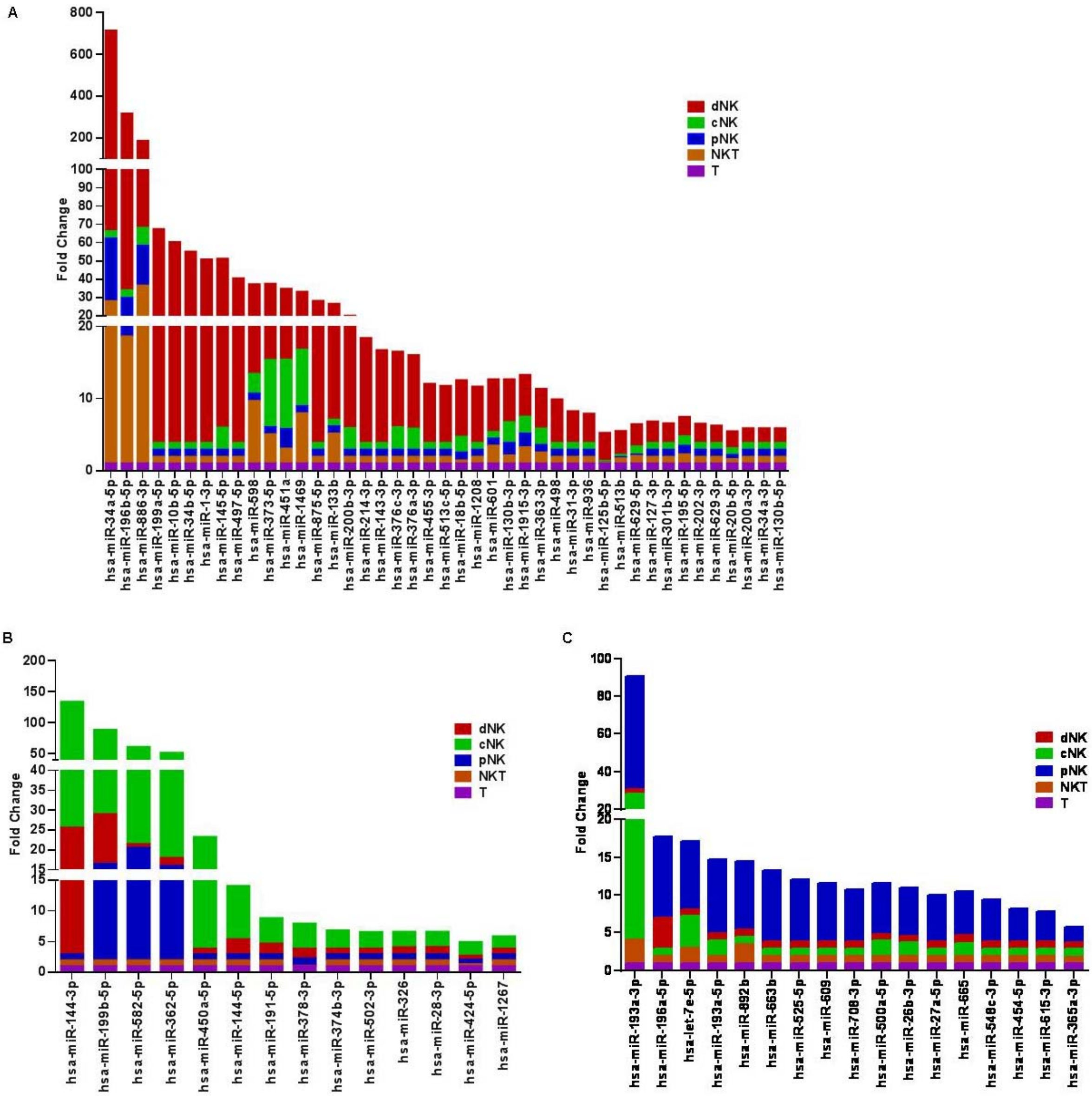

Supplementary Figure S1. miRNAs preferentially expressed in human primary NK cells. (A-C) miRNAs with a fold-change greater than two-fold in the dNK (A), cNK (B) and pNK (C) populations compared with the remaining four subsets.

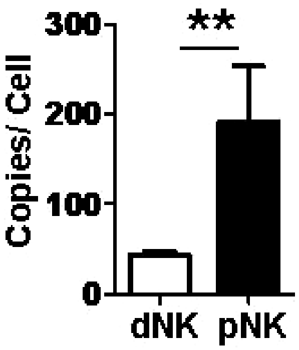

Supplementary Figure S2. The absolute expression of miR-362-5p in human primary NK cells. The Hairpin-it RT-PCR system (Genepharma, Shanghai, China) was used to determine miR-362-5p expression in various human NK cell populations. Total RNA samples were extracted using Trizol reagents. Reverse transcription of cDNA was performed from total RNA samples, using specific miRNA primers from the Hairpin-it miRNAs qPCR Quantitation Kit (Genepharma, Shanghai, China). Standard curves were generated by the software Rotor-Gene 6 (Corbett Research) based on the concentrations of synthetic miRNA standard. The automatic threshold function was applied to determine the threshold cycle (Ct) for each sample, and calculate the absolute number of copies with standard deviations. The copy number per cell was determined according to the number of NK cells that were used to produce the input RNA in PCR reactions. The data are representative of three independent experiments. $^{**}P < 0.01$(Student's *t*-test).

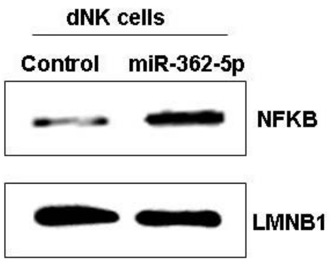

Supplementary Figure S3. MiR-362-5p affects NF-κB in human NK cells. Western blot analysis of the expression of nucleus NF-κB P65 protein in human dNK cells transfected with miR-362-5p mimics or a control miRNA. LMNB1 was used as the internal control. The data are representative of three independent experiments.

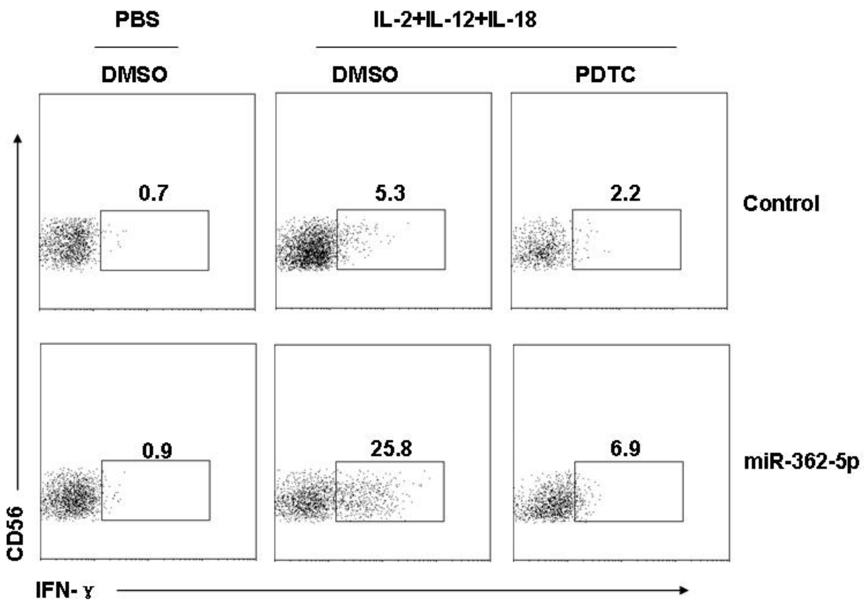

Supplementary Figure S4. miR-362-5p enhances IFN-γ production in human NK cells by activating the NF-κB pathway. Human primary uterine dNK cells were purified by magnetic activated cell sorting. After pretreatment with pyrrolidine dithiocarbamate (PDTC, NF-κB inhibitor, 50 μM) for 30 min, the expression of intracellular IFN-γ in cytokine-stimulated miR-362-5p-transfected dNK cells was then analyzed. The data are representative of three independent experiments.

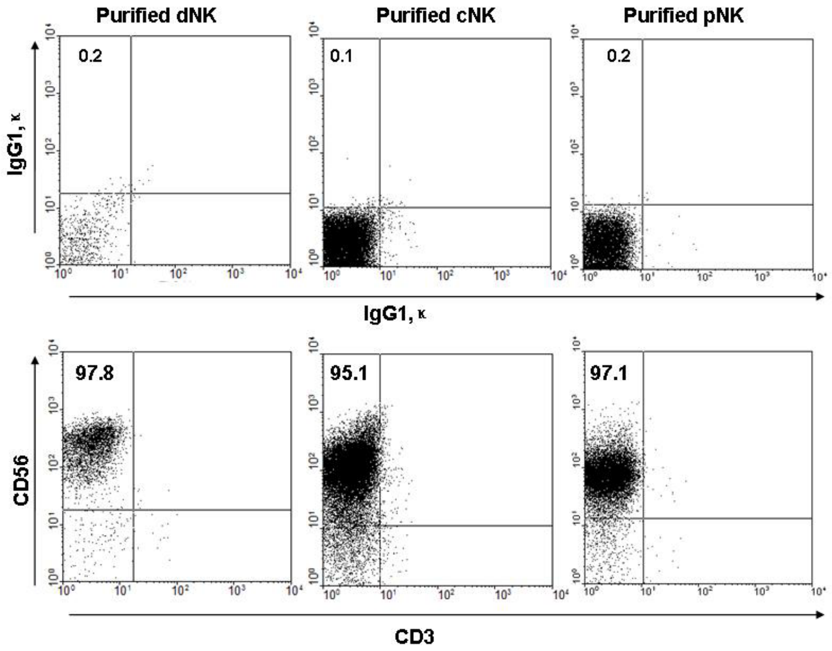

Supplementary Figure S5. The purity of the isolated human primary NK cells. Human decidual NK (dNK) cells were isolated from decidual samples using the MACS isolation system according to the manufacturer's instructions. Human cord blood NK (cNK) cells were isolated from cord blood mononuclear cells using MACS; human peripheral blood NK (pNK) cells were purified from peripheral blood mononuclear cells by sorting on a FACSAria (BD). The purified NK ($CD3^{-}CD56^{+}$) cells were stained with PE-Cy5 conjugated anti-CD3 and Alexa-647 conjugated anti-CD56 or isotype-matched negative control antibodies labelled with PE-Cy5 or Alexa-647 and analyzed by flow cytometry. The data are representative of three independent experiments.

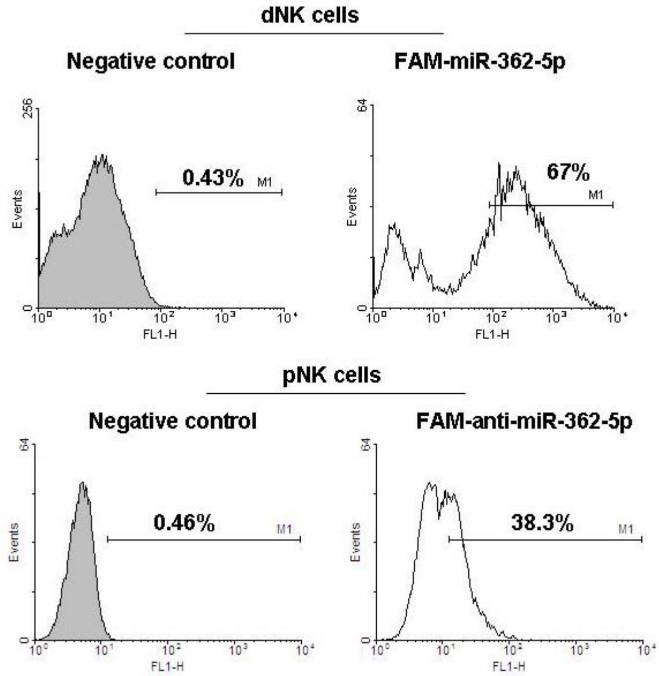

Supplementary Figure S6. The transfection efficiency of synthetic miRNAs in human primary NK cells. Flow cytometry analysis of FAM-positive human dNK or pNK cells transfected by nucleofection with FAM-labeled miR-362-5p mimics or inhibitors (anti-miR-362-5p). The data are representative of three independent experiments.

A. Figure 4E

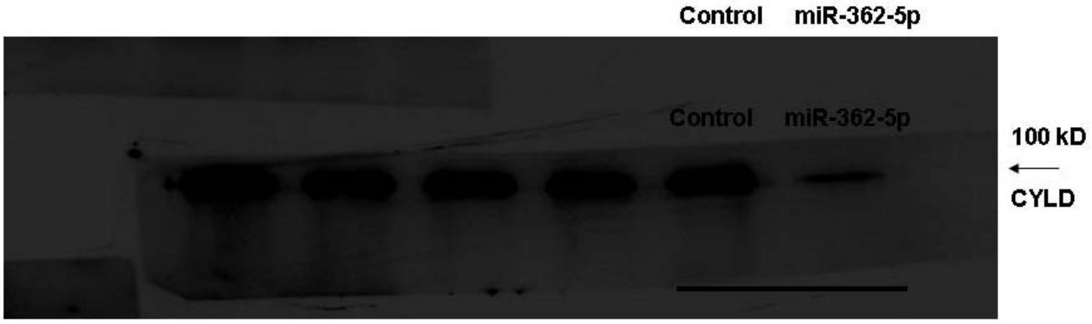

B. Figure 4E

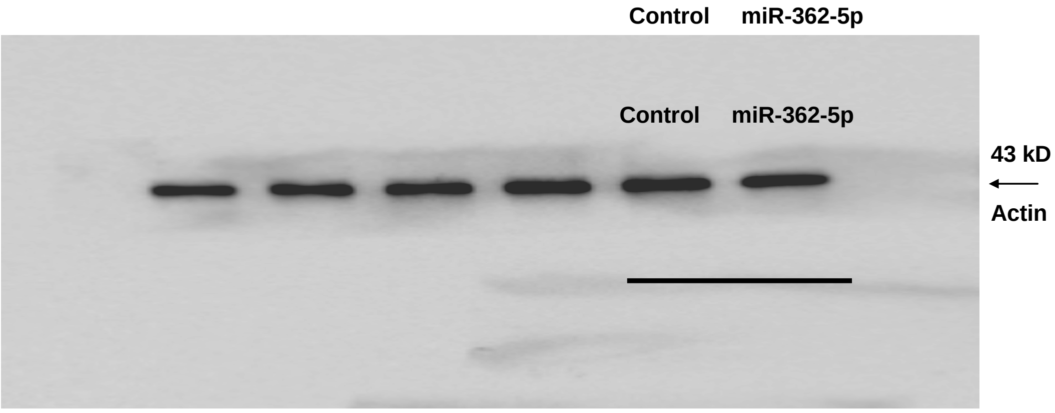

C. Figure 4G

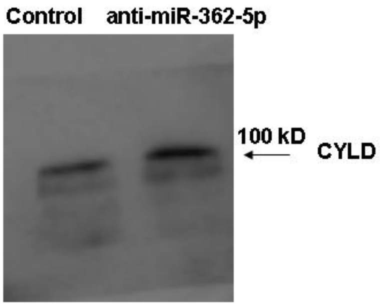

D. Figure 4G

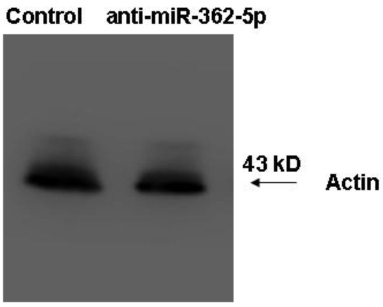

Supplementary Figure S7 Full-length images of the cropped blots presented in the main figures.

A and B: Full-length images in Fig. 4E demonstrate that miR-362-5p overexpression reduces CYLD expression in dNK cells. dNK cells were transiently transfected with miR-362-5p mimics or a control miRNA. Two days after transfection, dNK cells were collected and then analyzed by immunoblotting against CYLD. Actin was used as the internal control.

C and D: Full-length images in Fig. 4G illustrate that miR-362-5p knockdown increases CYLD expression in human primary pNK cells. Actin was used as the internal control.
